# P-1199. The burden of respiratory syncytial virus among Brazilian infants (BONSAI study): preliminary results

**DOI:** 10.1093/ofid/ofae631.1382

**Published:** 2025-01-29

**Authors:** Manoel Ribeiro, Rodrigo C da Silva, Karina Ribeiro, Aline Tolardo, Sheila Homsani, Rita Barata

**Affiliations:** Instituto Santa Casa de Educação, Pesquisa e Inovação, Sao Paulo, Sao Paulo, Brazil; Instituto Santa Casa de Ensino, Pesquisa e Inovação, São Paulo, Sao Paulo, Brazil; Sanofi, Sao Paulo, Sao Paulo, Brazil; Sanofi, Sao Paulo, Sao Paulo, Brazil; Sanofi, Sao Paulo, Sao Paulo, Brazil; Faculdade de Ciências Médicas da Santa Casa de São Paulo, Sao Paulo, Sao Paulo, Brazil

## Abstract

**Background:**

Respiratory syncytial virus (RSV) is a leading cause of global morbidity and mortality in children < 1 year. Recent data have shown the burden of the disease among full-term babies. RSV surveillance system in Brazil does not capture information about gestational age. We aimed to estimate the incidence of hospitalization due to RSV according to gestational age among infants in Brazil.

**Methods:**

Retrospective cohort study linking (deterministic and probabilistic, based on date of birth, city of residence, and mother´s name) the National Influenza Surveillance System (SIVEP-Gripe) and the National Information System on Live Births (SINASC). All children aged ≤24 months, living in São Paulo state, Brazil, admitted to hospitals clinically presenting with severe acute respiratory illness (SARI) tested for RSV from 2017 to 2019 were included. Incidence rates (IR) were calculated per 10,000 live births/year. Relative risk and corresponding 95% confidence intervals were estimated.

**Results:**

During the study period, 1,807,703 live births were registered in São Paulo state and 11,003 cases of SARI, with 1,801 cases positive for RSV (16.4%). We successfully linked 1,436 RSV cases with SINASC (79.7%). IR was 7.94/10,000 live births/year, peaking in April and May. Eighty-one percent of cases (n=1,166) were registered in full-term infants (37+ weeks) (IR=7.28 cases/10,000 live births/year). IR for infants born < 29, 29-31, 32-36 weeks (w) were 12.68, 19.74, and 12.84 cases/10,000 live births/year, respectively (RR, premature vs full-term=1.83; 95% CI 1.60-2.10). Forty-nine percent of full-term infants with RSV were admitted to ICU (n=570), while the figures for the other gestational age categories were: < 29 (76.5%), 29-31 (60%), 32-36 w (51.8%). The case-fatality rate was 0.8% overall (4% for premature and 0.4% for full-term infants).

**Conclusion:**

Our results showed that the burden of RSV among full-term infants is high (81% of hospitalized cases). Although the risk is higher among premature, a significant proportion of full-term infants can have unfavorable prognoses needing ICU admission, generating an overload of the healthcare system during seasonality. Preventive strategies for all infants are crucial to reduce the burden of RSV in Brazil.

**Disclosures:**

**Manoel Ribeiro, MD, PhD**, Sanofi: Grant/Research Support **Rodrigo C. da Silva, MSc**, Sanofi: Grant/Research Support **Karina Ribeiro, DDS, PhD**, Sanofi: Employee|Sanofi: Stocks/Bonds (Private Company) **Aline Tolardo, PhD**, Sanofi: Employee|Sanofi: Stocks/Bonds (Private Company) **Sheila Homsani, MD**, Sanofi: Employee|Sanofi: Stocks/Bonds (Private Company) **Rita Barata, MD, PhD**, Sanofi: Grant/Research Support

